# Bile acid dysregulation in sepsis: mechanisms, clinical implications, and future perspectives

**DOI:** 10.3389/fcimb.2026.1752527

**Published:** 2026-08-05

**Authors:** Jing Wang, Le Xia, Siyao Xu, Fei Wu, Suqin Shi, Yurong Wang, Yu Wei, Jiaqi Li, Xiaojie Zhang, Xiaoxiao Chen, Min Zhu, Jinqiang Zhuang

**Affiliations:** 1 Department of Emergency Intensive Care Unit (EICU), The Affiliated Hospital of Yangzhou University, Yangzhou University, Yangzhou, Jiangsu, China; 2 School of Nursing, Faculty of Medicine, Yangzhou University, Yangzhou, China; 3 Department of Intensive Care, The Affiliated Hospital of Yangzhou University, Yangzhou University, Yangzhou, Jiangsu, China; 4 The First School of Clinical Medicine, Faculty of Medicine, Yangzhou University, Yangzhou, China

**Keywords:** bile acids, biomarkers, gut–liver axis, microbiota, sepsis, translational medicine

## Abstract

Sepsis is a life-threatening syndrome caused by a dysregulated host response to infection and remains a major global health challenge because of its high morbidity, mortality, and limited targeted therapeutic options. Accumulating evidence suggests that bile acids (BAs), endogenous metabolites synthesized in the liver and modified by the gut microbiota, are involved in inflammation, barrier integrity, and metabolic homeostasis. Sepsis is associated with alterations in multiple aspects of BA homeostasis, including hepatic synthesis, transporter activity, microbial biotransformation, and enterohepatic circulation. These alterations may influence hepatic function, gut barrier integrity, and immune responses, although their precise role in sepsis progression remains incompletely understood. This review summarizes current advances in BA biology relevant to sepsis, discusses the interactions between BA dysregulation and the liver–gut–microbiota–immune axis, and evaluates the potential and current limitations of BA-related biomarkers and therapeutic strategies. Particular emphasis is placed on unresolved issues, including disease heterogeneity, causality, and clinical translation. By synthesizing current evidence and knowledge gaps, this review provides an updated perspective on BA dysregulation within the broader immunometabolic landscape of sepsis and highlights priorities for future research.

## 1 Introduction

Sepsis is a life-threatening syndrome caused by a dysregulated host response to infection and remains a major global health burden despite advances in supportive care (Fleischmann et al., 2016; Gotts and Matthay, 2016; Singer et al., 2016; Baykara et al., 2018; Buchman et al., 2020; Fleischmann-Struzek et al., 2020; Xie et al., 2020; Liu et al., 2022; Vincent, 2022). In addition to immune dysregulation, increasing evidence indicates that metabolic disturbances are closely associated with sepsis progression, organ dysfunction, and clinical outcomes (Michie, 1996; Nuyttens et al., 2024; Willmann and Moita, 2024). Among these, bile acids (BAs) have attracted growing attention because of their potential roles in linking metabolism, immunity, and host–microbiota interactions (de Aguiar Vallim et al., 2013; Sipka and Bruckner, 2014; Cai et al., 2022).

Current evidence suggests that sepsis may disrupt multiple aspects of BA homeostasis, and altered BA profiles may in turn modulate immune responses and organ function (Hao et al., 2017; Li et al., 2023). Whether these changes causally contribute to sepsis progression or largely reflect secondary consequences of systemic inflammation remains unclear, a distinction that is complicated by the inherent heterogeneity of sepsis (Davenport et al., 2016).

Several recent reviews have summarized aspects of BA metabolism, BA receptor signaling, gut microbiota-derived metabolites, intestinal barrier dysfunction, or sepsis-associated liver injury (Shi et al., 2023; Xu, 2025; Cheng et al., 2026; Zou et al., 2026). However, most existing discussions focus on individual components of the BA–microbiota–immune network or on BA biology in broader gastrointestinal and metabolic diseases, without a systematic focus on sepsis. Critical questions remain unresolved, including whether BA dysregulation acts as a causal driver or a downstream consequence of sepsis, how BA alterations interact with the heterogeneous clinical presentations of sepsis, and the translational feasibility of BA-based biomarkers or therapeutic interventions.

**Figure 1:**
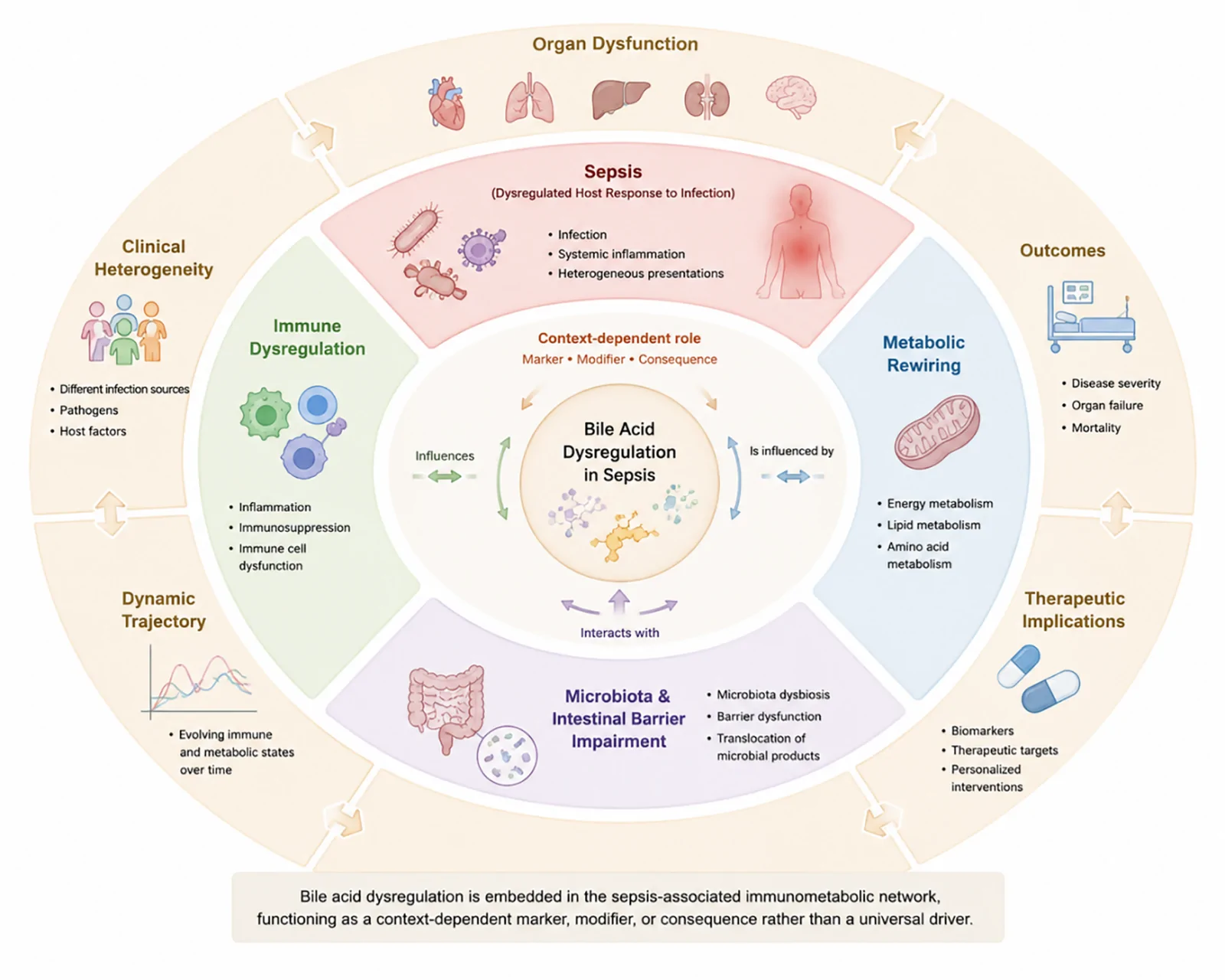
Conceptual framework of bile acid dysregulation within sepsis-associated immunometabolic dysfunction. Sepsis induces broad immunometabolic dysfunction involving immune dysregulation, metabolic disturbance, microbiota imbalance, and barrier impairment. Within this network, bile acid dysregulation should be viewed as an embedded, context-dependent component that may act as a marker, modifier, or consequence of disease, rather than a universal driver. This framework highlights the potential relevance of bile acid alterations to organ dysfunction, clinical heterogeneity, and translational applications in sepsis.

In this review, we provide a comprehensive overview of current evidence on BA metabolism in sepsis. We summarize how sepsis influences BA homeostasis and discuss the potential implications of BA alterations for immune regulation, organ dysfunction, and host–microbiota interactions. We further examine the emerging diagnostic and therapeutic relevance of BA-related pathways and discuss the major challenges that remain in translating these findings into clinical practice. By integrating current evidence within the broader framework of sepsis-associated immunometabolic dysfunction, this review aims to provide a balanced perspective on the role of BA dysregulation in sepsis and identify priorities for future research. A conceptual overview of these relationships is shown in **Figure 1**.

## 2 Bile acids and sepsis: biological background

### 2.1 Sepsis as a heterogeneous immunometabolic syndrome

Sepsis is a life-threatening syndrome characterized by a dysregulated host response to infection, resulting in immune imbalance, metabolic disturbances, and progressive organ dysfunction (Singer et al., 2016). Rather than representing a purely hyperinflammatory condition, sepsis is increasingly recognized as a dynamic process in which excessive inflammation and immunosuppression may coexist and evolve over time, jointly contributing to adverse clinical outcomes and multiple organ dysfunction (van der Poll et al., 2017; Nedeva et al., 2019).

In addition to immune dysregulation, sepsis is increasingly viewed as an immunometabolic syndrome (Pucillo and Vitale, 2020; Liu et al., 2023; Willmann and Moita, 2024). Systemic inflammation profoundly alters glucose, lipid, amino acid, and mitochondrial metabolism, leading to impaired energy homeostasis and organ dysfunction (Samra et al., 1996; Chioléro et al., 1997; Nuyttens et al., 2024; Zhang Y. et al., 2025). These metabolic alterations are closely intertwined with immune responses and may influence disease progression, treatment responses, and patient outcomes (Li et al., 2022; Xu et al., 2025; Deng et al., 2026).

A major challenge in sepsis research is its marked heterogeneity (Reddy et al., 2020; Scherger and Kalil, 2024; Antcliffe et al., 2025). Clinical manifestations, infection sources, causative pathogens, host characteristics, and immune-metabolic responses vary substantially among patients (Tietze et al., 2006; Motzkus and Luckmann, 2017; Denstaedt et al., 2018; Abe et al., 2019; Mendoza et al., 2022). Consequently, sepsis is increasingly considered a collection of biologically distinct syndromes rather than a single disease entity, which may partially explain the limited success of many targeted therapeutic strategies (Kellum et al., 2022; Zhang et al., 2024; Antcliffe et al., 2025).

Among the metabolic pathways affected during sepsis, bile acid metabolism has attracted increasing attention (Li et al., 2023; Gao and Arifuzzaman, 2026). Beyond their classical role in lipid digestion, bile acids function as signaling molecules involved in metabolic regulation, immune responses, and host–microbiota interactions (Thomas et al., 2008; Bao-Anh et al., 2025; Yang et al., 2025). Emerging evidence suggests that alterations in bile acid homeostasis are associated with sepsis severity, organ dysfunction, and clinical outcomes, making bile acid metabolism a potentially important component of sepsis-associated immunometabolic dysregulation (Wang J. et al., 2025; Zhang L. et al., 2025).

### 2.2 Overview of bile acid metabolism

BAs are cholesterol-derived metabolites synthesized in the liver and constitute the major end products of cholesterol catabolism (Danielsson and Sjövall, 1975). Under physiological conditions, BAs are maintained through a tightly regulated cycle involving hepatic synthesis, intestinal microbial transformation, and enterohepatic circulation (Russell, 2003).

In humans, primary BAs, mainly cholic acid (CA) and chenodeoxycholic acid (CDCA), are synthesized in hepatocytes through classical and alternative pathways (Hofmann, 2009). Following secretion into the intestine, primary BAs undergo extensive microbial biotransformation, generating secondary BAs such as deoxycholic acid (DCA) and lithocholic acid (LCA) (Dawson and Karpen, 2015; Guzior and Quinn, 2021; Zheng et al., 2024). These processes contribute to the diversity and biological activity of the circulating BA pool.

Under physiological conditions, most BAs are reabsorbed in the terminal ileum and returned to the liver through enterohepatic circulation, thereby maintaining BA homeostasis. Because BA synthesis, transport, microbial biotransformation, and recycling are tightly coordinated, disturbances in any of these processes can alter BA composition, abundance, and signaling properties (Percy-Robb and Collee, 1972; Joyce and Gahan, 2017; Zheng et al., 2024). Such alterations have been increasingly implicated in a variety of metabolic and inflammatory disorders, including sepsis, highlighting the importance of understanding BA homeostasis in disease pathophysiology (Wahlström et al., 2016; Lin et al., 2025).

### 2.3 Bile acid receptors: implications in sepsis

BAs exert signaling functions through several nuclear and membrane receptors, including farnesoid X receptor (FXR), Takeda G protein-coupled receptor 5 (TGR5), pregnane X receptor (PXR), constitutive androstane receptor (CAR), and vitamin D receptor (VDR) (Forman et al., 1995; Mangelsdorf and Evans, 1995; Makishima et al., 1999; Parks et al., 1999). These receptors regulate BA synthesis, transport, detoxification, and enterohepatic circulation while also influencing immune and metabolic processes (Li and Chiang, 2013).

FXR is considered the principal BA sensor and contributes to the maintenance of BA homeostasis through feedback regulation of BA synthesis and transporter expression (Goodwin et al., 2000; Holt et al., 2003; Kong et al., 2012). Beyond its role in BA metabolism, FXR has been implicated in pathways relevant to sepsis, including regulation of intestinal barrier integrity, inflammatory signaling, and host metabolic adaptation. Experimental studies have shown that FXR activation may attenuate gut microbiota dysbiosis, restore BA homeostasis, and improve barrier function in septic models (Qian et al., 2025). FXR signaling has also been associated with modulation of inflammatory responses and metabolic stress during endotoxemia and systemic inflammation (Ma et al., 2006; Prawitt et al., 2011; Chávez-Talavera et al., 2017; Geiger et al., 2021; Xu et al., 2022; He et al., 2025). However, whether these experimental findings translate into clinically meaningful benefits in human sepsis remains unclear.

TGR5 is a membrane BA receptor activated mainly by secondary BAs (Castellanos-Jankiewicz et al., 2021). It is mainly expressed in adipose and intestinal tissues and plays an important role in regulating inflammatory responses via cAMP-dependent signaling pathways (Watanabe et al., 2006; Thomas et al., 2009; Velazquez-Villegas et al., 2018). Experimental studies suggest that TGR5 activation may modulate innate immune responses and barrier function, processes that are frequently disturbed during sepsis (Jin et al., 2021; Reich et al., 2025). However, its clinical relevance in human sepsis remains uncertain.

Other bile acid-sensing receptors, including PXR, CAR, and VDR, contribute to bile acid detoxification, xenobiotic metabolism, and immune regulation (Staudinger et al., 2001; Zhang et al., 2004; Stedman et al., 2005; Miao et al., 2006). In addition, emerging evidence suggests that microbiota-derived bile acid metabolites may regulate host signaling through additional receptor pathways (Lin et al., 2025). Although their relevance in sepsis remains largely unexplored, these findings indicate that bile acid signaling extends beyond the classical FXR and TGR5 pathways.

**Figure 2:**
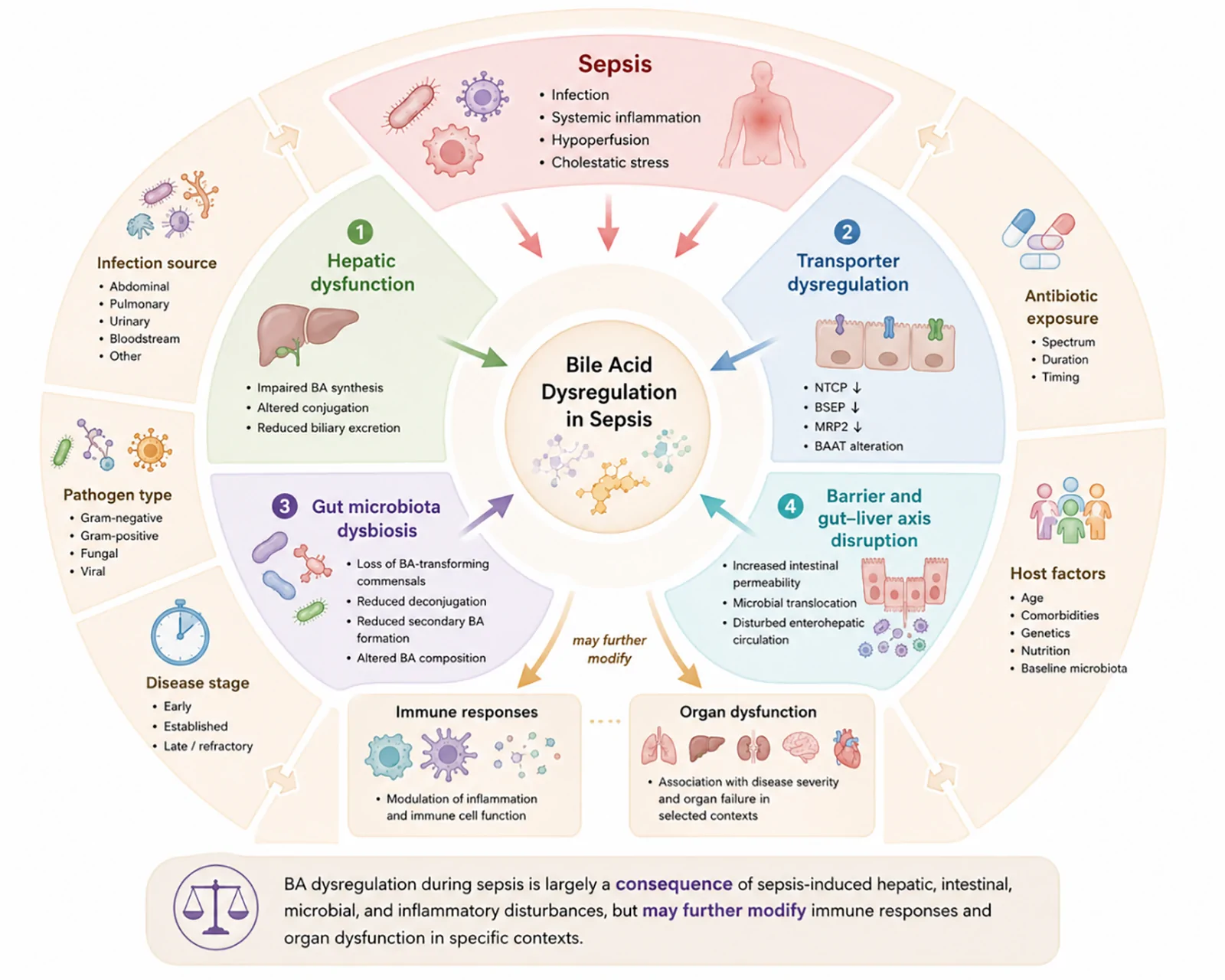
Mechanisms underlying bile acid dysregulation during sepsis. Sepsis disrupts bile acid homeostasis through hepatic dysfunction, transporter impairment, altered conjugation, gut microbiota dysbiosis, intestinal barrier injury, and disturbed gut–liver communication. These processes reshape bile acid levels and composition, indicating that bile acid dysregulation is largely embedded within sepsis-induced hepatic, intestinal, microbial, and inflammatory disturbances. In selected contexts, altered bile acid signaling may further modify immune responses and organ dysfunction.

Overall, BA receptor signaling provides a mechanistic link between BA metabolism and host immune-metabolic responses (Sun et al., 2021; Zheng et al., 2021; Song et al., 2025). However, current evidence is derived predominantly from experimental studies, and the role of these pathways in human sepsis remains incompletely understood. Their clinical relevance and therapeutic potential require further investigation. The major mechanisms underlying BA dysregulation during sepsis are summarized in **Figure 2**.

## 3 Alterations in bile acid homeostasis during sepsis

### 3.1 Hepatic dysfunction and altered BA synthesis, transport and conjugation

Clinical and experimental studies have consistently reported alterations in circulating BA profiles during sepsis. In patients with severe sepsis, plasma concentrations of specific BA species, including CDCA and taurodeoxycholic acid (TDCA), are significantly elevated at the time of diagnosis (Recknagel et al., 2012). Similar findings have been observed in experimental endotoxemia and septic shock, where total circulating BA levels were increased compared with healthy controls (Leonhardt et al., 2023). Animal studies further support these observations, as septic dogs exhibit significantly higher serum BA concentrations than non-septic controls (Baptista et al., 2022). Notably, some BA abnormalities appear before marked changes in conventional liver function indicators, suggesting that disturbances in BA metabolism may represent an early manifestation of hepatic and systemic dysfunction during sepsis (Recknagel et al., 2012).

As the central organ responsible for BA synthesis, conjugation, uptake, and biliary excretion, the liver plays a critical role in maintaining BA homeostasis. Consequently, sepsis-associated liver dysfunction can affect multiple steps of BA metabolism. Experimental and clinical studies have demonstrated impaired hepatocellular handling of bilirubin, BAs, and xenobiotics during sepsis, accompanied by abnormalities in BA conjugation and excretory processes (Recknagel et al., 2012). Importantly, these alterations may occur before the development of overt cholestasis, indicating that disruption of BA homeostasis can emerge early in the course of sepsis-associated liver injury.

Impaired BA transport represents another characteristic feature of hepatic dysfunction during sepsis. Physiologically, BA uptake and secretion depend on the coordinated activity of transporters located on the basolateral and canalicular membranes of hepatocytes. During sepsis and endotoxemia, reduced expression or altered function of key transporters, including sodium taurocholate cotransporting polypeptide (NTCP), bile salt export pump (BSEP), and multidrug resistance-associated protein 2 (MRP2), has been reported (Bolder et al., 1997). These changes may limit hepatic BA uptake and biliary secretion, thereby contributing to increased circulating BA concentrations and alterations in BA composition. In addition, suppression of bile acid-CoA:amino acid N-acyltransferase (BAAT) may impair BA conjugation and modify the relative abundance of conjugated and unconjugated BA species (Bolder et al., 1997).

The mechanisms underlying these alterations are likely multifactorial and may involve inflammatory signaling, cholestatic responses, and broader metabolic disturbances associated with sepsis (Bolder et al., 1997). Overall, available evidence supports a close association between sepsis-associated liver dysfunction and alterations in BA synthesis, transport, and conjugation. However, whether these changes actively contribute to disease progression or primarily reflect underlying hepatic and systemic dysfunction remains incompletely understood. Furthermore, most studies have been conducted in specific clinical populations or experimental models, making it difficult to determine whether similar patterns of BA alterations occur across different sepsis subtypes. Therefore, BA abnormalities should be viewed as one component of the complex metabolic disturbances observed during sepsis rather than as an independent or universal driver of disease progression.

### 3.2 Gut microbiota dysbiosis and BA biotransformation during sepsis

The gut microbiota is essential for shaping the composition and signaling properties of the BA pool (Guo et al., 2022). After primary BAs are synthesized and conjugated in the liver, a proportion enters the colon, where gut bacteria mediate deconjugation, dehydroxylation, oxidation, epimerization, and other modifications (Hofmann, 2009; Guzior and Quinn, 2021). These microbial reactions generate secondary BAs and other BA derivatives, thereby increasing the diversity of BA species and influencing their biological activity (Dawson and Karpen, 2015; Zheng et al., 2024).

During sepsis, the gut microbial ecosystem is frequently disturbed by systemic inflammation, intestinal hypoperfusion, antibiotic exposure, nutritional changes, and critical illness-related stress (Donskey, 2004; Zaborin et al., 2014; Sun et al., 2023). Clinical and experimental studies have reported reduced microbial diversity, loss of commensal anaerobes, and expansion of opportunistic pathogens in septic conditions (Haak et al., 2018; Tao et al., 2024). Because many BA-transforming bacteria are anaerobic commensals, sepsis-associated dysbiosis may alter the microbial capacity for BA biotransformation (Haak et al., 2018; Li Z. et al., 2024; Kullberg et al., 2025).

One important consequence of this dysbiosis is the potential disruption of microbial BA deconjugation and secondary BA formation. Under physiological conditions, bile salt hydrolase-producing bacteria deconjugate host-derived conjugated BAs, providing substrates for further microbial transformation. Subsequent reactions, including 7α-dehydroxylation, contribute to the generation of secondary BAs such as DCA and LCA (Jones et al., 2008; Ridlon and Hylemon, 2012; Joyce and Gahan, 2017; Song et al., 2019). When the abundance or activity of BA-transforming bacteria is altered during sepsis, the balance between primary, conjugated, unconjugated, and secondary BA species may shift. Such changes can modify BA receptor activation and downstream signaling, including FXR- and TGR5-related pathways.

Experimental evidence supports a link between sepsis-associated dysbiosis and BA metabolic disturbance. In septic mouse models, altered gut microbiota has been associated with activation of intestinal MyD88 signaling, suppression of the FXR/FGF15 feedback pathway, BA imbalance, and intestinal barrier injury (Qian et al., 2025). Fecal microbiota transplantation from septic mice to healthy mice reproduced several of these abnormalities, whereas probiotic intervention partially reversed microbial dysbiosis, restored BA-related signaling, and improved intestinal barrier function (Qian et al., 2025). These findings suggest that gut microbiota changes can influence BA homeostasis during sepsis, although most evidence remains derived from experimental models.

Therefore, interpretation of elevated BA levels during sepsis requires consideration of changes in BA composition rather than total BA concentrations alone. Variations in BA species, conjugation status, microbial biotransformation, and receptor interactions may contribute to distinct biological effects. This complexity also complicates efforts to define the precise role of BA alterations in sepsis.

Taken together, available evidence supports a close association between sepsis-associated gut microbiota dysbiosis and altered BA biotransformation (Li et al., 2023; Qian et al., 2025; Wu et al., 2026). However, whether these alterations actively influence disease progression or primarily reflect broader disturbances in intestinal ecology, hepatic function, antibiotic exposure, and host metabolism remains incompletely understood. Future studies should distinguish changes in total BA levels from changes in specific BA species and clarify how infection source, antimicrobial treatment, nutritional status, and host factors shape microbiota-dependent BA metabolism during sepsis.

### 3.3 Gut–liver axis dysfunction during sepsis

The gut–liver axis provides an anatomical and functional route through which intestinal signals, microbial products, metabolites, and BAs influence hepatic metabolism and immune responses (Tilg et al., 2022; Xu et al., 2023). Under physiological conditions, the intestinal barrier limits microbial translocation, while portal circulation enables bidirectional communication between the gut and liver (Pabst et al., 2023). BAs are involved in this communication by regulating gut microbial composition, enterohepatic circulation, and receptor-mediated signaling pathways (Li T. et al., 2024).

During sepsis, disruption of intestinal barrier integrity may alter this gut–liver communication (Zhang et al., 2022). Inflammatory injury to the intestinal epithelium can increase intestinal permeability, allowing endotoxins and microbial products to enter the portal and systemic circulation (Oami et al., 2024; Li et al., 2025). This process may expose the liver to increased inflammatory and microbial signals, thereby contributing to hepatic stress, cholestatic responses, and altered BA homeostasis. However, these changes should be understood as part of a broader sepsis-associated disturbance rather than as a BA-specific mechanism alone.

Experimental studies provide evidence that modulation of the gut–liver axis can influence sepsis-related outcomes. Sodium butyrate has been reported to enhance intestinal barrier function, regulate CD4^+^Foxp3^+^ regulatory T-cell balance, reduce endotoxin translocation, and improve sepsis-associated lung injury in CLP mice (Wei et al., 2024). In another study, FXR activation attenuated sepsis-associated dysbiosis, inhibited excessive intestinal MyD88 activation, restored BA metabolism and intestinal barrier function, and improved outcomes in experimental sepsis (Qian et al., 2025). These findings suggest that the gut–liver axis may represent an important interface linking intestinal barrier injury, microbial dysbiosis, BA signaling, and systemic inflammation during sepsis.

Nevertheless, current evidence remains largely experimental, and the clinical relevance of gut–liver axis modulation in human sepsis is not yet fully established. Therefore, this axis should be viewed as a context-dependent component of sepsis-associated metabolic and immune dysregulation rather than a universal therapeutic target.

### 3.4 Heterogeneity of BA alterations across sepsis contexts

A central limitation of the current literature is that BA dysregulation is often discussed as a general feature of sepsis, whereas sepsis itself comprises biologically distinct conditions with different infection sources, pathogens, host backgrounds, and treatment exposures.

Although altered BA homeostasis has been reported in sepsis, current evidence does not support a single, uniform BA pattern across all sepsis contexts. Available studies include heterogeneous populations and models, such as severe sepsis, septic shock, endotoxemia, and experimental polymicrobial sepsis (Recknagel et al., 2012; Baptista et al., 2022; Leonhardt et al., 2023; Qian et al., 2025). These settings differ substantially in disease severity, inflammatory stimulus, organ involvement, microbiota disruption, and treatment exposure. Therefore, BA abnormalities observed in one clinical cohort or experimental model should be interpreted within the specific context in which they were measured.

Infection source is a major but insufficiently examined variable. Gut-derived or abdominal sepsis may be more directly associated with intestinal barrier disruption, microbial dysbiosis, and altered enterohepatic circulation, whereas sepsis originating from the lung, urinary tract, bloodstream, or other sites may affect BA metabolism more indirectly through systemic inflammation, hepatic dysfunction, critical illness, or therapeutic interventions (Marshall et al., 2004; Jeganathan et al., 2017; Hecker et al., 2019). However, few studies have directly compared BA profiles across infection sources. As a result, it remains unclear whether abdominal sepsis has a distinct BA signature or whether BA changes mainly reflect shared features of critical illness and hepatic-metabolic stress.

Pathogen type represents another important source of uncertainty. Much of the mechanistic evidence linking sepsis to BA disturbance comes from endotoxemia or Gram-negative bacterial models, in which lipopolysaccharide-related signaling is prominent (Bolder et al., 1997; Leonhardt et al., 2023). These models are useful for studying inflammatory effects on BA transport and hepatic metabolism, but they may not fully capture the biology of Gram-positive, fungal, viral, or polymicrobial sepsis. Different pathogens activate different immune pathways and may exert distinct effects on intestinal barrier function, liver injury, gut microbiota composition, and microbial BA transformation (Cheung et al., 2021; Thomson et al., 2021). At present, whether pathogen-specific immune responses translate into distinct BA profiles remains largely unknown.

Host factors further complicate the interpretation of BA alterations. Pre-existing liver disease, cholestasis, obesity, diabetes, age, nutritional status, and antibiotic exposure can independently influence BA synthesis, transporter expression, microbial biotransformation, and enterohepatic circulation (So et al., 2020; Ren et al., 2024; Yang et al., 2024; Cadena Sandoval and Haeusler, 2025). These factors are common in critically ill patients and may confound the relationship between sepsis and circulating BA levels. Therefore, elevated BA concentrations during sepsis should not automatically be interpreted as a sepsis-specific metabolic signature; they may also reflect liver dysfunction, microbiota disruption, comorbid metabolic disease, or treatment-related effects.

Disease stage and sampling time are also important. BA profiles measured during early sepsis, septic shock, prolonged critical illness, or recovery may reflect different biological processes. Early changes may be related to acute inflammatory signaling and hepatic transporter dysfunction, whereas later changes may be shaped by cholestasis, prolonged antibiotic exposure, nutritional support, and sustained microbiota disruption (Crowell and Lang, 2021). Cross-sectional measurements are therefore limited in their ability to capture the temporal dynamics of BA metabolism during sepsis.

Taken together, the available evidence supports an association between sepsis and altered BA homeostasis, but it does not establish that BA dysregulation follows a universal pattern across all sepsis subtypes. Future studies should stratify patients according to infection source, pathogen type, disease stage, liver function, antibiotic exposure, nutritional status, and metabolic comorbidities. Longitudinal and subtype-specific analyses will be necessary to determine whether BA alterations represent general markers of critical illness, context-dependent features of specific sepsis phenotypes, or clinically useful indicators for prognosis and therapeutic decision-making.

## 4 Context-dependent contributions of BA dysregulation to sepsis pathophysiology

### 4.1 BA signaling and immune regulation

BAs may influence immune regulation during sepsis through both receptor-dependent and receptor-independent mechanisms. Rather than exerting a uniform effect, BA-mediated immune modulation appears to depend on BA species, concentration, receptor distribution, inflammatory context, and disease stage. This context-dependent feature is important for interpreting why some BA-related pathways show anti-inflammatory effects, whereas others may amplify inflammatory injury.

Experimental studies have shown that different BA species can exert divergent effects on inflammatory responses. CDCA has been reported to inhibit LPS-induced IL-6 secretion by macrophages, whereas DCA may promote TNF-α release from macrophages (Hao et al., 2017; Cao et al., 2021). Under septic inflammatory stress, toxic BAs such as DCA may also act as damage-associated molecular pattern-like signals by inducing prolonged calcium influx in inflammatory macrophages and cooperating with ATP to activate NLRP3 inflammasome signaling (Hao et al., 2017). These findings suggest that BA dysregulation should not be interpreted simply as protective or harmful; rather, the immunological consequences depend on the composition of the BA pool and the inflammatory microenvironment.

BA receptors provide another mechanism by which BAs may regulate inflammation. TGR5 activation suppresses pro-inflammatory cytokine release from LPS-stimulated Kupffer cells through the cAMP/PKA pathway (Keitel et al., 2008). In addition, TGR5 signaling has been associated with inhibition of NF-κB activation and reduced TNF-α production in macrophages (Pols et al., 2011; Wang et al., 2011; Yoneno et al., 2013; Wang et al., 2021). In septic models, activation of TGR5 has also been reported to reduce microglial activation and neutrophil infiltration in the hippocampus through the cAMP/PKA/CREB pathway, thereby alleviating sepsis-related cognitive impairment (Jin et al., 2021). These findings support a potential anti-inflammatory role of TGR5 signaling, particularly in immune cells and non-parenchymal tissues.

FXR signaling may also contribute to inflammatory regulation. FXR activation has been shown to attenuate NF-κB gene transcription induced by IL-1β in vascular smooth muscle cells and to downregulate interferon-γ-responsive genes in macrophages (Li et al., 2007; Renga et al., 2009). In septic mice, FXR activation alleviated liver injury by regulating BA homeostasis and inhibiting pro-inflammatory cytokine release, partly through the FXR/FGF15/FGFR pathway (Zhou et al., 2025). In addition, intestinal FXR activation may regulate gut microbiota composition, restore BA balance, and reduce intestinal inflammation in experimental sepsis (Qian et al., 2025). These observations suggest that FXR may link BA homeostasis, immune regulation, and gut–liver communication during sepsis.

Nevertheless, the available evidence remains limited in several respects. First, many findings are derived from *in vitro* experiments or animal models, and their applicability to human sepsis remains uncertain. Second, most studies focus on selected BA species or receptor agonists, whereas BA profiles in patients are dynamic and heterogeneous. Third, receptor activation may have different consequences depending on infection source, pathogen type, host liver function, microbiota status, and timing of intervention. Therefore, BA signaling should be viewed as a potential modifier of immune responses in selected contexts rather than as a universal driver of sepsis progression.

Taken together, current evidence indicates that BA signaling can regulate inflammatory pathways relevant to sepsis, particularly through DCA/NLRP3, TGR5/cAMP-PKA, and FXR/NF-κB-related mechanisms. However, whether these pathways determine clinical outcomes in human sepsis remains unresolved. Future studies should clarify which BA species, receptor pathways, disease stages, and patient subgroups are most relevant for BA-mediated immune regulation.

### 4.2 BA dysregulation, barrier integrity, and gut–liver crosstalk

The intestinal barrier and gut–liver axis represent important interfaces through which BA dysregulation may interact with sepsis-associated inflammation. Under physiological conditions, BAs participate in maintaining intestinal microbial balance, regulating epithelial barrier function, and supporting enterohepatic communication (Peterson and Artis, 2014; Albillos et al., 2020; Tilg et al., 2022). During sepsis, however, intestinal hypoperfusion, inflammation, microbiota dysbiosis, and hepatic dysfunction may jointly disturb this regulatory network (Bauer, 2022; Guo et al., 2022; Shi et al., 2023).

Experimental studies suggest that sepsis-induced intestinal injury can impair epithelial barrier integrity. In septic models, intestinal ischemia and hypoxia are associated with reduced expression of tight junction proteins such as Occludin and ZO-1, increased intestinal permeability, and enhanced translocation of endotoxins or microbial products (Qian et al., 2025). These changes may expose the liver and systemic circulation to increased inflammatory signals, thereby contributing to hepatic stress, altered BA metabolism, and systemic immune activation. However, this process should not be interpreted as a BA-specific mechanism alone, because barrier disruption in sepsis is also driven by inflammation, hypoperfusion, infection source, antibiotics, and critical illness-related stress.

Gut microbiota dysbiosis provides another route linking barrier injury and BA disturbance. The gut microbiota contributes to BA deconjugation and secondary BA formation through enzymes such as bile salt hydrolase, thereby shaping BA composition and receptor activation (Guzior and Quinn, 2021; Guo et al., 2022; Zheng et al., 2024). During sepsis, microbial diversity decreases, beneficial commensals are reduced, and potentially pathogenic bacteria may expand (Donskey et al., 2000; Donskey, 2004; Zaborin et al., 2014; Sun et al., 2023). Because many BA-transforming bacteria are anaerobic commensals, sepsis-associated dysbiosis may reduce the capacity for microbial BA transformation, leading to altered proportions of primary, conjugated, unconjugated, and secondary BAs (Guo et al., 2022; Collins et al., 2023; Lin et al., 2025).

Mechanistic evidence further supports an interaction between gut microbiota, BA signaling, and barrier function. In experimental sepsis, gut microbiota dysbiosis activates intestinal MyD88 signaling, suppresses the FXR-FGF15 pathway, disrupts BA homeostasis, and aggravates intestinal barrier dysfunction (Qian et al., 2025). Fecal microbiota transplantation from septic mice to healthy mice reproduced several of these abnormalities, whereas probiotic intervention partially reversed dysbiosis, restored BA-related signaling, improved tight junction integrity, reduced intestinal permeability, and decreased endotoxin translocation (Qian et al., 2025). These findings indicate that microbiota-dependent BA signaling may contribute to the regulation of barrier integrity in specific experimental settings.

BA receptor signaling may also participate in gut–liver communication. Intestinal FXR activation promotes FGF15/19-mediated feedback regulation of hepatic BA synthesis and may help maintain intestinal barrier function and microbial homeostasis (Holt et al., 2003; Inagaki et al., 2005; Schaap et al., 2009; Kong et al., 2012; Qian et al., 2025). In septic models, FXR activation was reported to restore BA metabolism, inhibit excessive intestinal MyD88 activation, improve barrier integrity, and reduce inflammatory injury (Qian et al., 2025). These findings suggest that the intestinal FXR–FGF15/19 axis may represent a mechanistic link between BA homeostasis, gut barrier function, and hepatic metabolic regulation during sepsis.

Nevertheless, several limitations should be emphasized. First, most available evidence comes from animal models, particularly abdominal or polymicrobial sepsis models, where gut barrier disruption and microbial translocation are prominent. Whether the same BA–barrier–gut–liver interactions occur in lung-derived, urinary tract-derived, bloodstream, viral, fungal, or other non-abdominal forms of sepsis remains unclear. Second, intestinal permeability, endotoxin translocation, and BA alterations may occur in parallel as consequences of systemic inflammation rather than forming a simple linear causal pathway. Third, antibiotic exposure, nutritional support, pre-existing liver disease, and baseline microbiota composition may strongly influence BA metabolism and barrier function in critically ill patients.

Taken together, current evidence supports a close association between BA dysregulation, intestinal barrier impairment, microbiota dysbiosis, and gut–liver axis dysfunction during sepsis. However, BA dysregulation should be viewed as one component of a broader intestinal–hepatic–immune disturbance rather than as an independent universal driver of barrier failure or endotoxin translocation. Future studies should determine whether specific BA species or receptor pathways can identify patients with gut-centered sepsis phenotypes and whether targeting BA signaling can provide clinically meaningful benefits beyond existing supportive and antimicrobial strategies.

### 4.3 BA dysregulation and organ dysfunction: cause, consequence, or both?

BA dysregulation is closely associated with organ dysfunction during sepsis; however, whether it acts as a primary driver, a secondary consequence, or a context-dependent modifier remains unclear. Elevated BA levels have been reported in septic patients, including CDCA and TDCA, which correlate with increased short-term mortality risk.

In adult patients with severe sepsis, plasma CDCA and TDCA on the day of diagnosis showed higher predictive efficacy for 28-day mortality than conventional liver function markers such as bilirubin, with combined detection further improving sensitivity and specificity (AUROC = 0.87) (Recknagel et al., 2012). Pediatric sepsis patients similarly exhibit higher total BA levels in non-survivors compared with survivors, highlighting the prognostic relevance of BA accumulation (Wang Y. et al., 2025).

Experimental evidence suggests that BA dysregulation can exacerbate organ injury through multiple mechanisms. In CLP-induced septic mice, FXR agonists ameliorated hepatic inflammation and corrected BA metabolic imbalance, reducing liver injury and improving overall outcomes (Wang J. et al., 2025; Zhou et al., 2025). TGR5 activation (e.g., via INT-777) attenuated microglial activation in the hippocampus, reduced neuroinflammation, and improved cognitive outcomes in septic rodents (Jin et al., 2021). Cardiac dysfunction in LPS-induced sepsis models was mitigated by FXR agonists through inhibition of ERK1/2-DRP signaling and preservation of mitochondrial function, suggesting organ-specific protective roles of BA signaling (Miao et al., 2024). Additionally, interventions such as probiotics, time-restricted feeding, or Golden bifid treatment improved multi-organ injury by modulating gut microbiota–BA interactions, thereby restoring barrier integrity, reducing oxidative stress, and alleviating organ dysfunction (Wu et al., 2024; Hu et al., 2025; Qian et al., 2025).

Despite these mechanistic insights, the causal role of BA dysregulation remains uncertain. Many findings derive from animal or *in vitro* models, and direct validation in septic human tissues is limited. Moreover, the effects of specific BA species, concentrations, receptor activation, and organ context vary substantially. For example, cardiomyocyte-specific TGR5 deletion in diabetic mice exacerbated lipid accumulation and cardiac dysfunction (Wang et al., 2024), but this effect has not been verified in septic myocardial injury models, highlighting the gap in translating mechanistic insights to clinically relevant sepsis.

Taken together, current evidence indicates that BA dysregulation acts as a context-dependent modifier of organ function in sepsis rather than as a universal causal driver. Its biological consequences are highly dependent on BA composition, receptor pathways (FXR, TGR5), organ-specific vulnerability, disease stage, infection source, host metabolic status, and comorbid conditions. This framework reconciles seemingly contradictory observations in the literature: while certain BA species and receptor activations confer anti-inflammatory or barrier-protective effects, others may exacerbate oxidative stress, inflammatory signaling, or organ injury depending on context. Future studies should aim to clarify the pathological significance of individual BA subtypes, validate mechanistic findings in septic *in vivo* models, and define which patient subgroups might benefit from targeted BA modulation. By integrating these context-dependent effects, this approach provides a more nuanced understanding of how BA dysregulation contributes to organ dysfunction in sepsis without overstating causality.

## 5 Clinical implications and translational challenges

### 5.1 BA alterations as potential biomarkers

BAs have been proposed as potential biomarkers for sepsis severity, prognosis, and risk stratification. Clinical studies indicate that elevated levels of specific BA species, including CDCA and taurodeoxycholic acid (TDCA), correlate with higher short-term mortality in adult patients with severe sepsis. On the day of diagnosis, plasma CDCA and TDCA showed higher predictive accuracy for 28-day mortality than conventional liver function markers such as bilirubin, and combined detection further improved sensitivity and specificity (AUROC = 0.87) (Recknagel et al., 2012). Pediatric patients similarly exhibit higher total BA levels in non-survivors compared with survivors, underscoring the prognostic relevance of BA accumulation (Wang Y. et al., 2025).

Beyond single-molecule measurements, multi-parametric approaches combining BA profiles, gut microbiota composition, microbial metabolites, and clinical severity scores may enhance patient stratification. For example, a prognostic model incorporating taurocholic acid concentration, Klebsiella abundance, butyric acid concentration, and SOFA score improved mortality risk assessment in hospitalized sepsis patients (Long et al., 2023). These integrated models suggest that BA alterations may serve as quantitative markers reflecting sepsis-associated metabolic and microbial disturbances. However, variability across infection sources, disease stages, host conditions, liver function, antibiotic exposure, and microbiota composition complicates direct translation to clinical practice.

**Figure 3:**
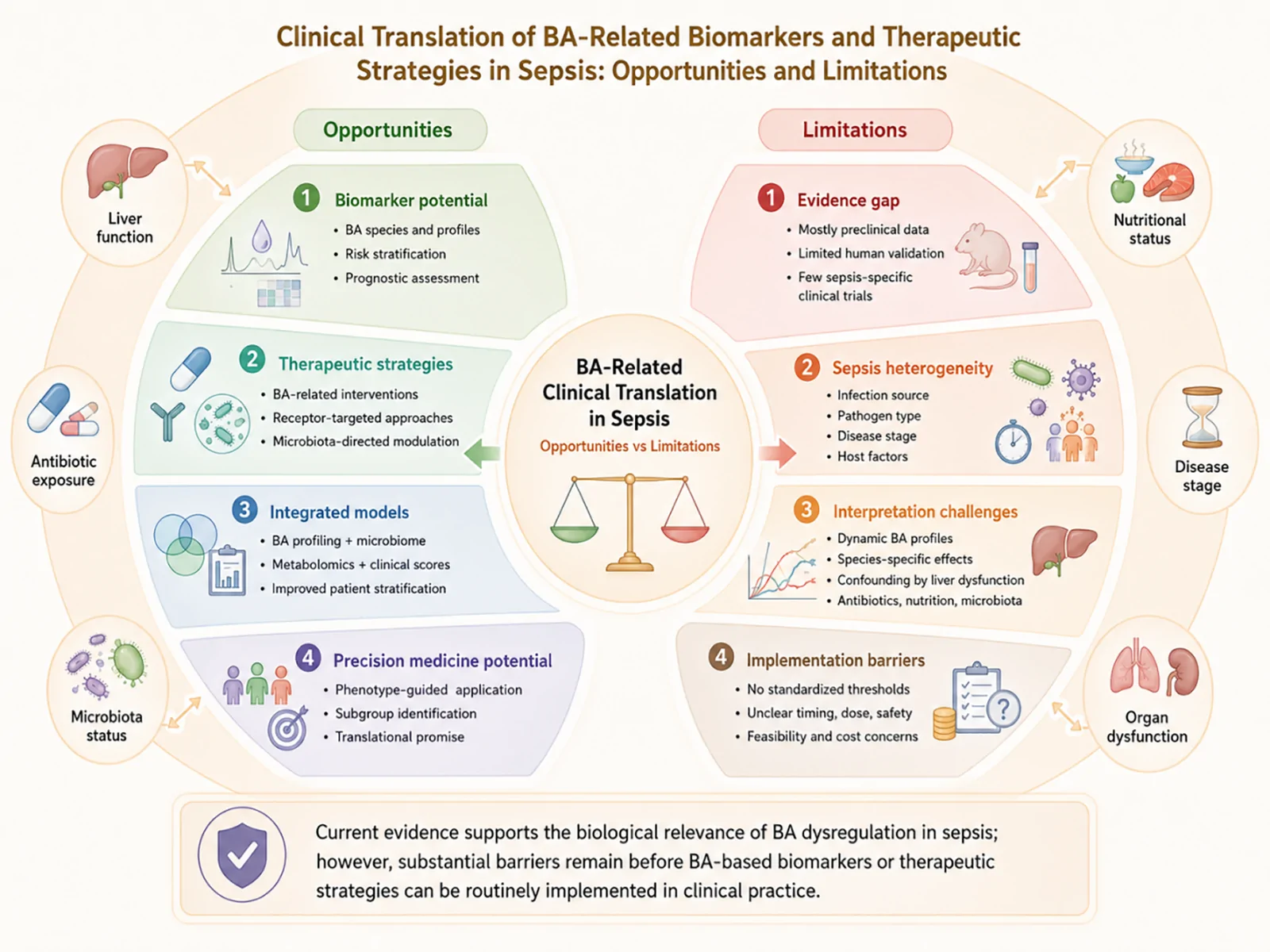
Clinical translation of bile acid-related biomarkers and therapeutic strategies in sepsis: opportunities and limitations. Bile acid dysregulation has emerged as a biologically relevant feature of sepsis with potential applications in biomarker development, therapeutic targeting, integrated multi-omics models, and patient stratification. However, substantial barriers remain before bile acid-based approaches can be translated into routine clinical practice, including limited clinical validation, sepsis heterogeneity, context-dependent bile acid alterations, confounding effects of liver dysfunction and treatment exposure, and practical implementation challenges. This image provides a balanced summary of the major translational opportunities and current limitations of bile acid-related strategies in sepsis.

Thus, BA-based biomarkers should currently be regarded as promising but not yet clinically established tools. Future studies should validate BA thresholds in large, multicenter cohorts, compare their added value with established biomarkers and clinical scores, and determine whether BA profiling can meaningfully improve risk stratification or therapeutic decision-making beyond existing approaches. The broader translational opportunities and limitations of BA-related approaches are summarized in **Figure 3**.

### 5.2 Therapeutic modulation of BA signaling

Therapeutic modulation of BA signaling has attracted attention as a potential strategy for mitigating sepsis-associated inflammation, barrier dysfunction, and organ injury. Current approaches mainly include receptor-targeted agents, such as FXR and TGR5 agonists, as well as microbiota-directed interventions that indirectly reshape BA metabolism. However, most evidence remains preclinical, and these strategies should be regarded as experimental or hypothesis-generating rather than clinically established therapies.

FXR agonists, particularly obeticholic acid (OCA), have shown protective effects in experimental sepsis models. OCA can activate FXR to regulate BA homeostasis, reduce unconjugated or toxic BA accumulation, and attenuate hepatic inflammatory responses (Wang J. et al., 2025). In septic or endotoxin-induced injury models, OCA has also been reported to inhibit NF-κB signaling, reduce oxidative stress, and improve kidney, liver, intestinal, and myocardial injury through mechanisms involving BSEP, ATF4-mediated autophagy, HIF-1α-regulated MDSC function, and ERK1/2-DRP-related mitochondrial protection (Xiong et al., 2017; Zhu et al., 2018; Jing et al., 2024; Miao et al., 2024; He et al., 2025). In addition, intestinal FXR activation promotes FGF15 signaling, supports epithelial repair, improves tight junction and mucus barrier integrity, reshapes gut microbiota composition, and improves survival in septic mice (Qian et al., 2025).

TGR5 activation represents another potentially relevant strategy. The TGR5 agonist INT-777 activates the TGR5-cAMP-PKA-CREB pathway, reduces microglial activation and neuroinflammation, and improves cognitive impairment and anxiety-like behaviors in septic rodents (Jin et al., 2021). Other BA-mediated TGR5-related mechanisms, including STAT3/A20-dependent suppression of NF-κB and MAPK signaling, have also been implicated in experimental sepsis models treated with Pien-Tze-Huang (Li B. et al., 2024). These findings suggest that TGR5 may modulate inflammatory responses in specific tissues, although clinical evidence in human sepsis remains lacking.

Microbiota-directed interventions may indirectly regulate BA signaling. In abdominal sepsis models, probiotic supplementation reduced intestinal MyD88 activation, restored the intestinal FXR-FGF15 axis, improved BA composition, strengthened tight junction integrity, reduced intestinal permeability, and decreased endotoxin translocation (Qian et al., 2025). Other gut microbiota-targeted or dietary interventions, including golden bifid treatment and time-restricted feeding, have also been reported to improve sepsis-associated myocardial or liver injury partly through microbiota-metabolite interactions (Wu et al., 2024; Hu et al., 2025). These approaches are attractive because they target the gut–microbiota–BA axis rather than a single receptor. Nevertheless, their efficacy may depend strongly on sepsis subtype, baseline microbiota composition, antibiotic exposure, intervention timing, and host metabolic status.

Overall, BA-targeted interventions show multi-organ protective potential in experimental sepsis, but several translational barriers remain. The efficacy and safety of most BA-related agents have not been validated in randomized clinical trials of sepsis patients. Moreover, adverse effects, drug interactions, optimal dosing, timing of intervention, and applicability across heterogeneous sepsis phenotypes remain unclear. Therefore, therapeutic modulation of BA signaling should be considered a promising but still exploratory strategy requiring rigorous preclinical and clinical validation. Representative BA-targeted therapeutic approaches and their reported effects in experimental sepsis are summarized in **Table 1**.

**Table 1:** BA-targeted therapeutic approaches and their reported effects in experimental sepsis.

Drug category	Drug name	Targets	Sepsis-protective effects	Potential limitations
FXR agonists	Obeticholic acid (OCA) (Wang J. et al., 2025)	FXR, BA transporters (NTCP, BSEP), FXR-downstream nuclear receptor SHP, NF-κB signaling pathway	1. Ameliorates septic liver dysfunction; 2. Regulates BA homeostasis; 3. Alleviates liver and ileal tissue injury, and maintains intestinal barrier integrity	1. New-onset severe pruritus; 2. Risk of hepatotoxicity
FXR agonists	OCA (Jing et al., 2024)	FXR, mitochondria, NF-κB signaling pathway	Improves septic kidney injury	1. New-onset severe pruritus; 2. Risk of hepatotoxicity
FXR agonists	OCA (Zhu et al., 2018)	FXR, NF-κB signaling pathway, NADPH oxidase, iNOS	Improves septic kidney injury	1. New-onset severe pruritus; 2. Risk of hepatotoxicity
FXR agonists	OCA (Xiong et al., 2017)	FXR, BSEP, ATF4, LC3II/I	Ameliorates septic liver injury	1. New-onset severe pruritus; 2. Risk of hepatotoxicity
FXR agonists	OCA (He et al., 2025)	FXR, HIF-1α, neonatal myeloid-derived suppressor cells (MDSCs)	1.Prevents neonatal sepsis; 2.Improves MDSCs function	1. New-onset severe pruritus; 2. Risk of hepatotoxicity
FXR agonists	OCA (Miao et al., 2024)	FXR, ERK1/2-DRP signaling pathway, mitochondria, antioxidant-related molecules (GPX1, SOD1, SOD2, NRF-2)	Ameliorates sepsis-associated myocardial injury	1. New-onset severe pruritus; 2. Risk of hepatotoxicity
FXR agonists	INT-747 (OCA) (Qian et al., 2025)	FXR, FGF15, intestinal barrier, gut microbiota	Alleviates intestinal injury induced by abdominal sepsis	1. New-onset severe pruritus; 2. Risk of hepatotoxicity
TGR5 agonists	INT-777 (Jin et al., 2021)	TGR5, cAMP-PKA-CREB signaling pathway	Improves sepsis-associated cognitive impairment	No reported limitations
BA derivatives	UDCA (Lou et al., 2025)	Nrf2/HO-1 pathway, NF-κB pathway, renal epithelial tight junction proteins (ZO-1, Ezrin)	Prevents septic kidney injury	1. Mild gastrointestinal symptoms; 2. Rare skin reactions
BA derivatives	UDCA (Golden et al., 2018)	EGFR (epidermal growth factor receptor), COX-2, ERK pathway	Prevents intestinal-derived sepsis	1. Mild gastrointestinal symptoms; 2. Rare skin reactions
BA derivatives	UDCA (He et al., 2023)	STING pathway, PANoptosis-like cell death	Ameliorates septic lung injury	1.Mild gastrointestinal symptoms; 2.Rare skin reactions
BA derivatives	UDCA (Milivojac et al., 2025)	NF-κB signaling pathway	1. Ameliorates septic lung injury; 2. Reduces serum levels of creatine kinase, lactate dehydrogenase, high-sensitivity cardiac troponin I, and homocysteine	1. Mild gastrointestinal symptoms; 2. Rare skin reactions
BA derivatives	CDCA (Milivojac et al., 2025)	NF-κB signaling pathway	Ameliorates septic lung injury	1. Diarrhea; 2. Mild elevation of transaminases
BA derivatives	TDCA (Chang et al., 2018)	Splenic granulocytic myeloid-derived suppressor cells	Improves septic kidney injury	No reported limitations
Camptothecin-BA conjugates	G2 (Xiao et al., 2020)	NF-κB signaling pathway	Ameliorates septic liver injury	No reported limitations
Other targeted agents	Pien Tze Huang (PZH) (Li B. et al., 2024)	TGR5, STAT3, A20, NF-κB pathway, MAPK pathway	Ameliorates sepsis	No reported limitations
Other targeted agents	Probiotics (Qian et al., 2025)	FXR-FGF15 axis, intestinal epithelial cell Myd88, FXR, BA composition	Improves sepsis-associated gut microbiota dysbiosis and alleviates sepsis	No reported limitations

### 5.3 Practical and translational limitations

Despite growing interest in BA-targeted approaches, several important challenges currently limit their clinical translation.

First, most evidence supporting BA-targeted interventions remains preclinical. FXR agonists, TGR5 agonists, probiotics, and other BA-modulating strategies have demonstrated beneficial effects in experimental sepsis models, including improvements in inflammation, barrier function, and organ injury (Jin et al., 2021; Miao et al., 2024; Qian et al., 2025; Wang J. et al., 2025; Zhou et al., 2025). However, these findings are derived predominantly from animal studies, whereas randomized controlled trials in septic patients remain scarce. Furthermore, potential interactions between BA-targeted therapies and standard sepsis treatments, including antibiotics, fluid resuscitation, vasopressors, and organ-support therapies, have not been systematically evaluated (Huang et al., 2019; Li B. et al., 2024).

Second, mechanistic understanding remains incomplete. Although experimental studies suggest that specific BA species may exert either protective or detrimental effects through FXR-, TGR5-, or inflammasome-related pathways (Li et al., 2007; Keitel et al., 2008; Renga et al., 2009; Huang et al., 2019; Cao et al., 2021; Jin et al., 2021), the causal relevance of these mechanisms in human sepsis remains uncertain. For example, Mendelian randomization analyses suggest opposing associations between different BA species and sepsis outcomes, with TDCA showing a potential protective association, whereas GCA and TCDCA were associated with increased mortality risk (Pan et al., 2026). However, these observations remain largely associative and lack validation in septic *in vivo* models. Similarly, several organ-protective functions attributed to FXR and TGR5 signaling have not been confirmed across different sepsis phenotypes or infection settings (Jin et al., 2021; Wang et al., 2024).

Third, the clinical utility of BA-based biomarkers remains uncertain. Although CDCA, TDCA, and total BA levels have shown prognostic value in both adult and pediatric sepsis cohorts (Recknagel et al., 2012; Long et al., 2023; Wang Y. et al., 2025), important questions remain regarding optimal thresholds, reproducibility across populations, and incremental value over established biomarkers and clinical scoring systems. In addition, BA profiles are influenced by numerous factors, including liver function, antibiotic exposure, nutritional status, microbiota composition, age, and metabolic comorbidities (Samra et al., 1996; Jones et al., 2008; Kullberg et al., 2025). These confounders may complicate interpretation and limit the generalizability of BA-based risk stratification approaches.

Finally, practical implementation presents additional challenges. The dynamic nature of BA metabolism means that measurements obtained at a single time point may not accurately reflect disease progression. Moreover, several proposed translational strategies rely on characterization of gut microbiota composition or microbiota-derived metabolites (Long et al., 2023), which may be difficult to assess in real time in critically ill patients. The feasibility, cost-effectiveness, and clinical utility of integrating BA profiling, microbiome analysis, and multi-omics approaches into routine sepsis management therefore remain uncertain.

Taken together, current evidence supports the biological relevance of BA dysregulation in sepsis, but substantial barriers remain before BA-based biomarkers or therapies can be routinely implemented in clinical practice. Addressing these limitations will require large multicenter cohorts, longitudinal sampling strategies, mechanistic validation in clinically relevant models, and carefully designed sepsis-specific clinical trials.

### 5.4 Future directions

Future research should move beyond describing associations between BA dysregulation and sepsis outcomes toward defining when, where, and in whom BA-related pathways are clinically meaningful. Given the heterogeneity of sepsis, longitudinal studies are needed to characterize dynamic BA profiles across different disease stages, infection sources, pathogen types, liver function states, antibiotic exposures, and nutritional conditions. Such studies would help determine whether BA alterations represent general markers of critical illness, context-dependent indicators of specific sepsis phenotypes, or actionable targets for intervention.

Mechanistic studies should further clarify the causal relevance of individual BA species and receptor pathways in clinically relevant sepsis models. Current evidence suggests that FXR, TGR5, MyD88, NLRP3 inflammasome signaling, and gut microbiota-dependent BA transformation may participate in sepsis-associated inflammation, barrier dysfunction, and organ injury (Keitel et al., 2008; Hao et al., 2017; Cao et al., 2021; Jin et al., 2021; Qian et al., 2025). However, the protective or injurious effects of specific BA subtypes remain incompletely verified *in vivo*, and several non-inflammatory functions of FXR and TGR5 have not been systematically tested under septic conditions (Wang et al., 2024; Pan et al., 2026). Future work should therefore distinguish whether BA changes are drivers, modifiers, or consequences of organ dysfunction in different sepsis contexts.

From a translational perspective, BA-based biomarkers require rigorous validation before clinical use. Although CDCA, TDCA, and total BA levels have shown prognostic value in adult and pediatric sepsis cohorts (Recknagel et al., 2012; Wang Y. et al., 2025), their thresholds, reproducibility, and incremental value over established biomarkers or clinical scoring systems remain uncertain. Integrating BA profiling with microbiome, metabolomic, transcriptomic, and clinical data may improve risk stratification, but such models should be tested in multicenter cohorts and assessed for practical feasibility in critically ill patients.

Therapeutic studies should also proceed in a stepwise and phenotype-aware manner. FXR/TGR5 agonists, probiotics, and microbiota-related interventions have shown protective effects in experimental sepsis models (Li B. et al., 2024; Wu et al., 2024; Hu et al., 2025; Qian et al., 2025; Wang J. et al., 2025). However, future studies need to define optimal timing, dosing, safety, and interactions with standard sepsis treatments. Rather than assuming broad efficacy across all patients, BA-targeted interventions may be more rationally evaluated in subgroups with gut-centered sepsis, liver dysfunction, cholestatic features, microbiota dysbiosis, or distinct BA signatures.

Overall, future research should integrate longitudinal clinical sampling, mechanistic validation, multi-omics modeling, and carefully designed sepsis-specific trials. This approach may clarify the position of BA dysregulation within the broader immunometabolic network of sepsis and determine whether BA-related biomarkers or interventions can provide clinically meaningful benefits beyond existing diagnostic and therapeutic strategies.

## 6 Conclusion

BA dysregulation is increasingly recognized as an important component of sepsis-associated immunometabolic disturbance, linking hepatic dysfunction, gut microbiota dysbiosis, intestinal barrier injury, and immune regulation. However, current evidence does not support viewing BA alterations as a universal causal driver of sepsis progression. Rather, BA dysregulation should be interpreted as a context-dependent process that may reflect disease severity, modify inflammatory and metabolic responses, and contribute to organ dysfunction in selected settings.

The clinical potential of BA-related biomarkers and therapeutic strategies remains promising but not yet established. Specific BA species and BA-related signaling pathways may improve risk stratification or provide therapeutic targets, particularly when integrated with microbiome, metabolomic, and clinical data. Nevertheless, patient heterogeneity, infection source, disease stage, liver function, antibiotic exposure, and limited clinical validation continue to constrain translation. Future studies should prioritize longitudinal profiling, mechanistic validation, multicenter biomarker studies, and carefully designed sepsis-specific trials to determine whether BA-focused approaches can offer clinically meaningful benefits beyond existing diagnostic and therapeutic strategies.
